# Knowledge, Attitudes, and Practices of HIV-Positive Adolescents Related to HIV/AIDS Prevention in Abidjan (Côte d'Ivoire)

**DOI:** 10.1155/2020/8176501

**Published:** 2020-12-27

**Authors:** Azagoh-Kouadio Richard, Yeboua Kossonou Roland, Yao Kouassi Christian, Kouassi-Kouadio Amenan Cécile, Aholi Jean Michel, Cissé Lacina, Asse Kouadio Vincent

**Affiliations:** ^1^Paediatric Ward of University Teaching Hospital of Angré, Abidjan, Côte d'Ivoire, 28 BP 1530, Abidjan 28, Côte d'Ivoire; ^2^Paediatric Ward of University Teaching Hospital of Bouaké, Côte d'Ivoire, 01 BP 1174, Bouaké 01, Côte d'Ivoire; ^3^Paediatric Ward of University Teaching Hospital of Treichville, Abidjan, Côte d'Ivoire, 01 BP V3, Avbidjan 01, Côte d'Ivoire

## Abstract

**Introduction:**

In sub-Saharan Africa, many adolescents living with HIV adopt behaviors and practices at risk of transmitting this infection. The aim of the study was to assess the knowledge, attitudes, and practices of HIV-positive adolescents regarding the transmission of HIV for the prevention of this disease.

**Methods:**

Knowledge Attitude Practical Survey (KAP) conducted from June 20 to August 22, 2018 in pediatrics at the University Hospital of Treichville. It included consenting HIV-positive adolescents followed up in the voluntary testing counseling unit. The variables studied related to sociodemographic aspects, knowledge, attitude, and practice relating to the transmission of HIV. The analysis was descriptive.

**Results:**

The active queue was 349 children, including 210 adolescents. Fifty adolescents (22 boys and 28 girls) participated in the study, a participation rate of 24%. The average age of the participants was 16 years (extreme 10 and 19 years). The respondent was an orphan (38%), a secondary school (58%), and separated living parents in 42%. He stated that he did not know he was infected in 62%, and that he had sex with at least one partner in 54%. He knew the modes of transmission and the means of prevention in 72% of the cases. He knew that an HIV-positive adolescent could transmit the disease in 68%, and that HIV/AIDS was incurable in 40%. 42% of respondents said that an infected person on ARVs was contagious. Twenty-nine respondents who had a partner said they had unprotected sex in 58%. All of the adolescents surveyed said that they did not talk to friends and family about HIV.

**Conclusion:**

The level of knowledge, attitude, and practice of HIV-positive adolescents regarding HIV/AIDS transmission is insufficient. We suggest setting up a therapeutic HIV/AIDS education program for these adolescents.

## 1. Introduction

Adolescents are human beings between the ages of 10 and 19 [1]. In 2015, this age group accounted for 16% of the world's population, or 1.2 billion people [2]. Adolescence is a stage of life during which individuals have unique psychological, social, and health needs [3]. The rapid physical and hormonal development of this period is sometimes accompanied by a desire for self-discovery, an emerging sense of autonomy, separation from caregivers, and assertion of independence, as well as a search for recognition and acceptance, which can lead to risk behaviors [4, 5]. In the literature, several demographic, behavioral, and social factors have been associated with the occurrence of these risk behaviors in adolescents. These include being male and lack of supportive parent-child relationships. Other factors such as living in rural and underdeveloped areas, substance use (drugs, alcohol), peer influence, and nonparticipation in sexual education programs have been identified [6]. Adolescent sexual risk behavior is a global public health problem [7, 8]. Unprotected sex exposes the adolescent to intentional or unintentional trauma, unwanted pregnancy, and sexually transmitted infections (STIs) such as human immunodeficiency virus (HIV) infection [1]. With regard to HIV infection, UNICEF estimates that approximately 700 adolescents aged 10-19 years are infected with HIV every day, or one infected adolescent every two minutes. Some 360,000 adolescents will die of HIV/AIDS-related illnesses between 2018 and 2030 [9]. In Côte d'Ivoire, the prevalence of HIV infection has decreased due to antiretroviral therapy. This prevalence has reduced from 3.7% in 2012 [10] to 2.6% in 2018 [5], and it is about 460,000 people.

Of these HIV-infected people, 31,000 were children, including 16,000 HIV-positive adolescents and youth [11]. Adolescents living with HIV depend entirely on their families for care. However, many adolescents are noncompliant with treatment due to socioeconomic, cultural, individual, and environmental factors [3, 12]. The consequences of noncompliance are abandonment of care, adoption of risky behaviors, persistence of viral load, occurrence of complications that increase the risk of death, and an increase in new infections [13, 14]. Optimizing adherence to antiretroviral therapy (ART) has been shown to prolong the lives of patients living with HIV and improve their quality of life, which are priority health indicators [15, 16]. Adolescents' lack of adherence to ART is likely due to a lack of knowledge, attitudes, and inappropriate practices about HIV/AIDS infection. However, this has not yet been studied among adolescents in Côte d'Ivoire. The objective of the study was to assess the knowledge, attitudes, and practices of HIV-positive adolescents regarding HIV transmission for the reduction of morbidity and mortality of this disease.

## 2. Methods

This was a descriptive and analytical cross-sectional study carried out in the voluntary consultation and screening unit (VCT) of the pediatric service of the Treichville University Hospital from June 20 to August 22, 2018. This unit was opened on June 1 2005, as part of the scale-up recommended by the WHO, in the fight against HIV/AIDS. The study population consisted of adolescents received in consultation in the unit during the study period. The adolescent inclusion criteria were positive HIV status and verbal/written informed consent of the adolescent and the parent or legal guardian. Not included were all HIV-infected adolescents who refused to participate in the study.

The study sample was drawn up during the adolescents' visits to the programmed unit twice a week, Wednesday and Thursday between 8 am and 12 noon. The variables studied related to sociodemographic characteristics (gender, age, education, etc.) and the adolescent's knowledge, attitude, and practice relating to HIV/AIDS infection. Knowledge is defined as the set of information that adolescents have about HIV/AIDS to prevent its transmission. As for attitude, it is the set of convictions and perceptions of the respondents that underlie their behavior relating to the prevention of the transmission of HIV/AIDS. And the practice represents the set of acts and gestures that adolescents take to prevent the transmission of HIV/AIDS. The information was gathered from the adolescents through a structured interview. Each adolescent was interviewed only once during the course of the investigation. The data collection instrument consisted of a set of standardized closed and open-ended questions. There were eight (8) questions for the sociodemographic characteristics of adolescents, eleven (11) questions for the knowledge of respondents on HIV/AIDS, and seven (7) questions for the attitudes and practices of adolescents living with HIV regarding prevention of transmission. For the validation of the data collection instrument, a pretest was carried out in the voluntary screening center (CDV) of the pediatric service of Cocody University Hospital over a week. This is because of the similarities of the patients with those of the place of our study. To carry out the study, the agreement of the Medical and Scientific Direction of the Treichville University Hospital and the informed consent of the adolescent and the parents or legal guardians were required. The data collected were processed and analyzed on the EPI info 7 software. The analysis was descriptive and consisted of calculating the workforce and determining means and proportions. The quantitative variables were analyzed as an average. Qualitative variables were expressed as a proportion. For each variable analyzed, the performance threshold was arbitrarily set at 75%.

## 3. Results

### 3.1. Participation Rate and Sociodemographic Characteristics of Respondents

During the study period, 210 adolescents infected with HIV had consulted the voluntary counseling and testing unit (VCT) of the pediatric service of the Treichville teaching hospital. Of these, 50 adolescents participated in the study, a participation rate of 24%. The respondent was male in 22 cases (44%) and female in 28 cases (56%) for a sex ratio of 0.78. In 76% of the cases, the adolescent's age was between 14 and 19 years. Thirty-eight percent of adolescents were orphans. Among orphans, only 6/19, or 32%, are in school. HIV1 was involved in all cases. The other sociodemographic aspects of adolescents are described in Table 1. Not all of the adolescents interviewed were in a therapeutic education program.

### 3.2. Knowledge of Respondents

Teenagers interviewed said they had all heard of HIV/AIDS. The information channel was school (37%), television (27%), health personnel (18%), family (7%), friends (4%), the internet (4%), and newspapers (3%). For those surveyed, 78% and 22% of HIV infections were transmissible and noncommunicable, respectively. In 94% of the cases, the adolescent interviewed knew at least one mode of transmission and one means of preventing HIV. The route of HIV transmission cited was in descending order: the sexual route (32%), blood transfusion (24%), contaminated sharp object (22%), and cross-contamination of HIV from mother to child (16%). Regarding the means of prevention mentioned, it was a condom (42%), abstinence (22%), and fidelity (20%) (Table 2). Regarding the fact that an HIV-positive adolescent could transmit HIV to those around or partner, 68% of those surveyed answered yes against 32% of no. According to the adolescents interviewed, an apparently healthy person could and could not transmit HIV in 44% and 56%, respectively. They also claimed that HIV/AIDS was curable and incurable in 40% and 60%, respectively. Respondents said in 58% of cases that a person on ARVs could be contagious while 22% argued that not.

### 3.3. Attitude, Practice, and Suggestions of Adolescents towards HIV Infection

Of the 50 teens surveyed, 27 (54%) said they had a partner. The partner was unique in 48% and multiple in 52%. Respondents who have partners all had unprotected sex. Twenty percent of adolescents who had unprotected sex spoke to their partners about their status. He communicated their status with their comrade in 20% of the cases. The suggestion of adolescents surveyed living with HIV is in Table 3.

## 4. Discussion

This descriptive and analytical cross-sectional study carried out in the Voluntary Consultation and Screening Unit (VCT) of the pediatric service of the Treichville University Hospital aims to assess the knowledge, attitudes, and practices of HIV-positive adolescents relating to HIV transmission for reducing the disease burden of this condition. It emerges from the study that the adolescents surveyed knew at least one mode of transmission and one means of HIV prevention in 91% and 81%, respectively, but had the majority of behaviors and practices at risk of HIV transmission. These results should be qualified because the study is monocentric, and the information was gathered during an interview conducted with the adolescent in the presence of the parent or legal guardian. For adolescents who do not speak French, we used an interpreter. A transcription bias cannot be excluded. Despite the methodological limitation, the study elicits the following discussion points in terms of participation rate, sociodemographic characteristics, knowledge, attitude, and practice of the adolescents surveyed.

### 4.1. At the Level of Participation Rate and Sociodemographic Characteristics of the Respondents

The study reveals a 24% participation rate. This result is lower than those reported by Rotermann in 2012 in Canada with 81% for 2003 and 72% for 2009-2010, respectively [17]. Meena et al. [18] in Delhi, India, in 2015 also found a participation rate of 82.1%. However, Labra et al. [19] in 2017 in Quebec, Canada, reported a participation rate of 20.1% among students. Indeed, the methodological bias linked to hospital studies and the refusal of adolescents to participate in the study could explain the low participation rate. The majority of adolescents in the cohort living with HIV are in a vulnerable situation (orphan, separated living parents), and the education level of adolescents is low in the study. This finding was reported by UNAIDS in 2008, estimating that 15 million children are orphaned by HIV and 80% of these orphans live in sub-Saharan Africa [20]. In Côte d'Ivoire, an estimated 320,000 children between the ages of 0 and 17 have been orphaned by HIV/AIDS [11]. The rate of orphans of at least one parent found in the study is relatively high (38%) and remains similar to that observed in 2003 by M'pemba. [21] in Brazzaville (39%). In contrast, Atakouma et al. [22] in Togo reported an orphan rate of 80% in 2007. This confirms that AIDS is a disease providing for infected or affected orphans. These precarious living conditions, family, and social vulnerability (socioeconomic difficulties, death of a parent, foster care, etc.) affect HIV-positive children [23] and generate major family upheavals. The low level of education of the adolescents in the study was found in the study by Kigombola et al. [24] in the Kisarawe district of Tanzania in 2007. These adolescents infected with HIV/AIDS in vulnerable situations are dependent on other members of their family with the risk of stigmatization and irregular follow-up hampering proper care. The study finds that 62% of adolescents living with HIV ignore their status and have multiple partners with multiple sex. Elkington et al. [25] noted a propensity for multipartnership since 88.9% of the adolescents in the study (United States) reported having many partners (no data on the simultaneous or consecutive nature of multi partnership was provided). For Krantz et al. [26], boys have more partners, younger relationships, and more unprotected sex than girls. These teens in full sexual maturation want to discover everything in sexuality. The study found that all adolescents are infected with HIV1. Azagoh et al. [27] reported in 2018 to the pediatric emergencies of the CHU Bouaké (Côte d'Ivoire) a proportion of 92.8% and 7.2%, respectively, for HIV 1 and HIV 2. D'Almeida et al. [28] in Benin in 2013 objectified HIV 1 in 100% of cases. These results could be explained by the fact that among the three types of HIV infection, HIV 1 is the most widespread in sub-Saharan Africa.

### 4.2. At the Level of Knowledge of our Respondents

The study reveals that the communication channel is school and television in almost two thirds of the cases. The predominant role of the media (television, radio, and newspapers) as a source of information in the acquisition of knowledge, noted in our study, has also been noted in several studies [19, 29, 30]. The majority of adolescents living with HIV know at least one mode of transmission and one means of preventing HIV, but have behaviors and practices at risk of HIV transmission. These results are similar to those of several authors who noted a good knowledge of those interviewed on HIV/AIDS [29–31]. Forty-four percent of teens surveyed say that someone in apparent good health can transmit HIV, compared to 56% who say the opposite. Touré et al. [30] in Abidjan found that the majority of adolescents (89%) knew that a person in apparent good health could be carrying the AIDS virus and transmit it. Denial of infection is common. Many young people do not believe they are threatened by HIV. In Benin and Malawi, for example, most young people of both sexes know how to transmit AIDS and how to prevent it, but many are convinced that they cannot be infected with HIV [32]. In Haiti, almost two thirds of young girls aged 15 to 19 with an active sex life do not think they are at risk of being infected with HIV [33]. They claim in our study that HIV/AIDS is curable and incurable, respectively, in 40% and 60%. Respondents said in 58% of cases that a person on ARVs can be contagious while 22% contends the opposite.

### 4.3. At the Level of Attitudes and Practices

In the study, only 10% of respondents (5/50) discussed HIV with their parents in the family. The majority of adolescents (40/50) or 80% did not communicate with their friends and did not practice awareness raising on HIV/AIDS. This could be explained by ignorance of their status and fear of stigma [34]. The study found that 54% of these adolescents (27/50) have at least one partner. Among these 27 adolescents, 48% claim to have a single partner, 41% two partners, and 11% of multiple partners. In the study by Touré et al. [30], all respondents in sexual activities had at least two sexual partners, and 26.6% had more than 5 sexual partners. Amon et al. [35] reported 23.4% of teens aged 10 to 19 who reported being sexually active and 42.0% of them reported having had multiple sexual partners in the past 12 months. This could be explained by the fact that the majority ignored their status and by the need to hide their illness for fear of being stigmatized. These risky behaviors are a factor in the spread of the disease [36]. All 27 teenagers interviewed with at least one partner had unprotected sex in the study. Although the condom is approximately 90% effective in preventing HIV transmission, its impact can be limited by inconsistent use and low use in those most at risk as reported by Hearst et al. [37]. Tavory et al. [38] found in rural Malawi that using a condom meant a risky, less serious, and less intimate partner. Furthermore, even when people believe that condom use is appropriate, wise, or even a matter of life and death, the statement that condom use makes on a relationship usually trumps all other meanings. In the study by Igham et al. [39], roughly equal proportions of respondents knew their partner's sexual status or assumed their sexual status so that they did not see the importance of using a condom during sexual intercourse. Fear of being stigmatized and rejected by the sexual partner (s) could be a factor in not using condoms. Access to the condom could also be an obstacle to its use. Indeed, Amon et al. [35] reported in their study that the overall prevalence of condom use among them was 39.2%. The proportion who used a condom the last time they had sex was higher among those who knew they could get a condom if they wanted than among those who did not. This same observation was made by Meekers et al. [40]. This study shows that many adolescents find that access to condoms is more difficult in public sector outlets than in private sector outlets, because providers in the public sector tend to question adolescent behavior, unlike the latter. Women are reluctant to ask their friends for condoms because they fear that their friends will chat about them. Babalola et al. [41] found that urban residence and age negatively influence primary sexual abstinence and positively affect condom use. Living in the same household as the father tends to protect girls from early sexual experimentation but has no significant effect on boys. Other reasons have been reported in the literature such as the meaning of sex as an act of procreation so that condom use is usually only negotiated in certain short-term relationships [42].

### 4.4. Suggestions

All adolescents (100%) made suggestions for preventing the transmission of HIV/AIDS. More than half of them (54%) wanted a definitive solution to be found by discovering HIV vaccine prevention (Table 3). Current progress in clinical and biomedical research has certainly contributed to improving the health of people infected with the AIDS virus, but as long as no vaccine is marketed, prevention remains the only means of combating AIDS. It is based on information and education campaigns for the population, which make it essential to have AIDS prevention elements in school curricula [43].

## 5. Conclusion

From our study, it appears that the level of knowledge, attitude, and practice of HIV-positive adolescents regarding the transmission of HIV/AIDS are insufficient, with multiple factors contributing to increasing the risk of contamination in young people: risky sexual behavior, particularly multisexual partnership and nonuse of condoms. Hence, the need for a therapeutic education program on HIV/AIDS for these adolescents who encourage abstinence, the reduction of the number of sexual partners, and the use of condoms for sexually active adolescents. Follow-up must necessarily be accompanied by substantial psychosocial support, particularly for the most vulnerable, adolescents and orphans.

## Figures and Tables

**Table 1:** Distribution by sociodemographic characteristics of HIV-positive adolescents (*N* = 50).

Variables	Numbers (*n*/*N*)	Percentage
Sex		
Female	28/50	56
Male	22/50	44
Age in year		
[10–13]	12/50	24
[14–16]	16/50	32
[17–19]	22/50	44
Study level		
Not educated	6/50	12
Primary	12/50	24
Secondary	29/50	58
Superior	3/50	6
Social situation		
Orphan	19/50	38
Married parents	21/50	42
Separated parents	29/50	58
Knowledge of HIV status		
Yes	19/50	38
No	31/50	62
Existence of partner		
Yes	27/50	54
No	23/50	46

**Table 2:** Distribution of respondents by knowledge of HIV/AIDS (*N* = 50).

Knowledge	Numbers (*n*/*N*)	Percentage
Transmission mode		
Sexual	16/50	32
Blood	12/50	24
Contaminated sharp object	11/50	22
Cross-section (mother-child)	8/50	16
Do not know	3/50	6
Prevention methods		
Condom	21/50	42
Abstinence	11/50	22
Do not know	10/50	20
Fidelity	8/50	16
Healing of HIV/AIDS		
Yes	30/50	60
No	20/50	40

**Table 3:** Distribution of respondents according to the suggestions relating to the prevention of the transmission of HIV AIDS (*N* = 50).

Answers	Number (*n*/*N*)	Percentages
Vaccine prevention	27/50	54
Awareness on prevention of HIV transmission	21/50	42
Healthy life	14/50	28
Creation of NGOs to support HIV-positive adolescents	11/50	22

## Data Availability

The [sociodemographic and clinical] data used to support the findings of this study are available upon request from the corresponding author.

## References

[1] WHO *Adolescent development*.

[2] UNAIDS (2016). *UNAIDS 2016 Estimates*.

[3] Adejumo O. A., Malee K. M., Ryscavage P., Hunter S. J., Taiwo B. O. (2015). Contemporary issues on the epidemiology and antiretroviral adherence of HIV-infected adolescents in sub-Saharan Africa: a narrative review. *Journal of the International AIDS Society*.

[4] Gardner M., Steinberg L. (2005). Peer influence on risk taking, risk preference, and risky decision making making in adolescence and adulthood: an experimental study. *Developmental Psychology*.

[5] Chein J., Albert D., O'Brien L., Uckert K., Steinberg L. (2011). Peers increase adolescent risk taking by enhancing activity in the brain's reward circuitry. *Developmental Science*.

[6] Do H. N., Nguyen D. N., Nguyen H. Q. T. (2020). Patterns of Risky sexual Behaviors and associated factors among Youths and adolescents in Vietnam. *International Journal of Environmental Research and Public Health*.

[7] Kirby D., Laris B. A., Rolleri L. (2005). *Impact of sex and HIV education programs on sexual behaviors of youth in developing and developed countries. FHI Working Paper Series No. WP05-03*.

[8] Bryan A. D., Schmiege S. J., Magnan R. E. (2012). Marijuana use and risky sexual behavior among high-risk adolescents: trajectories, risk factors, and event-level relationships. *Developmental Psychology*.

[9] UNICEF AIDS: 360, 000 adolescents will die between 2018 and 2030. https://www.unicef.fr/article/sida-360-000-adolescents-mourront-entre-2018-et-2030.

[10] MSLS/INS Demographic and health survey and with multiple indicators in Ivory Coast 2011-2012: prevalence of HIV. https://www.dhsprogram.com/pubs/pdf/HF46/HF46.pdf.

[11] UNAIDS *Country factsheets: Côte d’Ivoire 2018*.

[12] Tran B. X., Hoang C. L., Tam W. (2020). A global bibliometric analysis of antiretroviral treatment adherence: implications for interventions and research development (GAPRESEARCH). *AIDS Care*.

[13] Maskew M., Bor J., MacLeod W., Carmona S., Sherman G., Fox M. P. The youth treatment bulge in South Africa: increasing numbers, inferior outcomes among adolescents on ART.

[14] Judd A., Chappell E., Doerholt K., Galli L., Giaquinto C., Gibb D. Longterm trends in mortality and AIDS-defining events among perinatally HIV infected children across Europe and Thailand.

[15] Vu G. T., Tran B. X., Hoang C. L. (2020). Global research on quality of life of Patients with HIV/AIDS: is it Socio-Culturally Addressed? (GAPRESEARCH). *International Journal of Environmental Research and Public Health*.

[16] Hudelson C., Cluver L. (2015). Factors associated with adherence to antiretroviral therapy among adolescents living with HIV/AIDS in low- and middle-income countries: a systematic review. *AIDS Care*.

[17] Rotermann M. (2012). Sexual behaviour and condom use of 15- to 24-year-olds in 2003 and 2009/2010. *Health Reports*.

[18] Meena J. K., Verma A., Kishore J., Ingle G. K. (2015). Sexual and reproductive health: knowledge, attitudes and perceptions among young unmarried male residents of Delhi. *International Journal of Reproductive Medicine*.

[19] Labra O., Lacasse A., Gingras-Lacroix G. (2017). Attitudes and knowledge of university students with regard to HIV/AIDS. *Social Service*.

[20] UNAIDS Report on the global AIDS epidemic 2008. https://www.unaids.org/sites/default/files/media_asset/jc1736_2008_annual_report_en_1.pdf.

[21] M’pemba Loufoua-Lemay A. B., Nzingoula S. (2003). AIDS at the CHU of Brazzaville: experience of the pediatrics department "big children". *Bulletin de la Société de Pathologie Exotique*.

[22] Atakouma D. Y., Tsolenyanu E., Gbadoe A. (2007). Primary results of antiretroviral treatment among HIV/AIDS infected children in Lome (Togo). *Archives de Pédiatrie*.

[23] Nicolas J. (1999). *Child HIV and AIDS, what quality of life?*.

[24] Kigombola A., Gotora G. (2007). Knowledge, attitude and practices on HIV/AIDS, its transmission and prevention among primary school pupils in rural Kisarawe. *DMSJ*.

[25] Elkington K., Bauermeister J., Brackis-Cott E., Dolezal C., Mellins C. A. (2009). Substance use and sexual risk behaviors in perinatally human immunodeficiency virus-exposed youth: roles of caregivers, peers and HIV status. *The Journal of Adolescent Health*.

[26] Krantz S., Lynch D., Russel J. (2002). Gender-specific profiles of self-reported adolescent HIV risk behaviors. *The Journal of the Association of Nurses in AIDS Care*.

[27] Azagoh K. R., Yao K. C., Bénié A. C. (2018). Prevalence and factors associated with the transmission of HIV/AIDS infection to pediatric emergencies at the center Hospitalier Universitaire (CHU) in Bouaké. *RAMUR*.

[28] d'Almeida M., Sagbo G., Lalya F. (2013). Profile of HIV-infected children at the National University Hospital of Cotonou (CNHU). *Le Mali Médical*.

[29] Ombotto S., Nonga B. N., Ntone F., Ambassa G., Zonk D. A. (2016). Knowledge, attitude and behavioral practices regarding HIV/AIDS infection and prevention among rural students in Cameroon, Attitude and Behavioral Practices Regarding HIV/AIDS Infection and Prevention among Rural Students in Cameroon. *Journal Community Medical Health Education*.

[30] Touré B., Koffi K., Kouassi-Gohou V., Kokoun E., Angbo-Effi O., Koffi N. M. (2005). Knowledge, attitudes and practices of middle and high school students in Abidjan in the face of HIV/AIDS. *Tropical Medicine*.

[31] Vijayageetha M., Narayanamurthy M. R., Vidya G. S., Renuka M. (2016). Knowledge and attitude on HIV/AIDS among adolescent school children in urban Mysuru, Karnataka, India: a cross sectional study. *International Journal of Community Medicine and Public Health.*.

[32] National Program to Fight AIDS and STIs in Benin (1999). *Multicentric study on the factors that determine the differences in levels of HIV infection in Africa*.

[33] UNAIDS (2001). *HIV/AIDS in Africa*.

[34] Naar-King S., Wright K., Parsons J. T. (2006). Healthy choices: motivational enhancement therapy for health risk behaviors in HIV-positive youth. *AIDS Education and Prevention*.

[35] Exavery A., Lutambi A. M., Mubyazi M. G., Kweka K., Mbaruku G., Masanja H. (2011). Multiple sexual partners and condom use among 10 - 19 year-olds in four districts in Tanzania: what do we learn?. *BMC Public Health*.

[36] Sallah E. D., Grunitzky-Bekele M., Bassabi K., Dodzro K., Sadzo A., Balogou A. K. (1999). Sexual behavior, knowledge and attitudes of students at the University of Benin (Togo) towards AIDS and sexually transmitted diseases. *Health*.

[37] Hearst N., Chen S. (2004). Condom promotion for AIDS prevention in the developing world: is it working?. *Studies in Family Planning*.

[38] Tavory I., Swidler A. (2009). Condom semiotics: meaning and condom use in rural Malawi. *American Sociological Review*.

[39] Ingham R., Woodcock A., Stenner K. (1991). Getting to know you: young people's knowledge of their partners at first intercourse. *Journal of Community and Applied Social Psychology*.

[40] Meekers D., Ahmed G., Molatlhegi M. T. (2001). Understanding constraints to adolescent condom procurement: the case of urban Botswana. *AIDS Care: Psychological and Socio-medical Aspects of AIDS/HIV.*.

[41] Babalola S., Awasum D., Quenum-Renaud B. (2002). The correlates of safe sex practices among Rwandan youth: a positive deviance approach. *African Journal of AIDS Research*.

[42] Bond V., Dover P., Sheldon S. (1997). The impact of HIV/aids on education: a review of literature and experience. UNESCO, Section for Preventive Education, 1994. *Health Transition Review*.

[43] Sheldon S. (1994). *The Impact of HIV/Aids on Education: A Review of Literature and Experience*.

